# Environmental remediation at vegetable marketplaces through production of biowaste catalysts for biofuel generation

**DOI:** 10.1038/s41598-023-31687-5

**Published:** 2023-03-28

**Authors:** T. Sathish, R. Saravanan, Melvin Victor Depoures, B. Palanikumar, M. Rajasimman, S. Rajkumar

**Affiliations:** 1 Department of Mechanical Engineering, Saveetha School of Engineering, SIMATS, Chennai, Tamil Nadu India; 2 Department of Civil Engineering, Sethu Institute of Technology, Virudhunagar, Tamil Nadu India; 3 Department of Chemical Engineering, Annamalai University, Annamalai Nagar, Chidambaram, India; 4 Department of Mechanical Engineering, Faculty of Manufacturing, Institute of Technology, Hawassa University, Hawassa, Ethiopia

**Keywords:** Energy science and technology, Engineering

## Abstract

Large quantities of vegetable biowaste are generated at marketplaces, usually in highly populated locations. On the other hand, nearby markets, hotels, and street shops generate much cooking oil waste and dispose of them in the sewage. Environmental remediation is mandatory at these places. Hence, this experimental work concentrated on preparing biodiesel using green plant wastes and cooking oil. Biowaste catalysts were produced from vegetable wastes and biofuel generated from waste cooking oil using biowaste catalysts to support diesel demand and Environmental remediation. Other organic plant wastes such as bagasse, papaya stem, banana peduncle and moringa oleifera are used as heterogeneous catalysts of this research work. Initially, the plant wastes are independently considered for the catalyst for biodiesel production; secondary, all plant wastes are mixed to form a single catalyst and used to prepare the biodiesel. In the maximum biodiesel yield analysis, the calcination temperature, reaction temperature, methanol/oil ratio, catalyst loading and mixing speed were considered to control the biodiesel production. The results reveal that the catalyst loading of 4.5 wt% with mixed plant waste catalyst offered a maximum biodiesel yield of 95%.

## Introduction

Based on worldwide environmental considerations, the research concentrates on producing alternative fuels. Alternative fuel production focuses on reducing pollution and fuel cost^1^. Studied and produced the bioethanol using sweet potato, pineapple and jackfruit wastes. For their study, pineapple waste produced 0.090% ethanol, sweet potato waste produced 0.079%, and jackfruit waste produced 0.45% ethanol. Recently, the heterogeneous catalyst is a significant role in biodiesel production for better activity, minimum cost and easy separation. Heterogeneous catalysts offered a faster reaction rate in biodiesel production^2,3^.

Avoiding environmental pollution and reducing crude oil usage by introducing ethanol/biofuel through cassava flour. This investigation studied the yeast concentrations and fermentation time on ethanol production. Using De-oiled rice bran (DORB) hydrolysate by Saccharomyces cerevisiae MTCC 4780 for ethanol production, the maximum ethanol yield is attained as 9.68% with a temperature of 30 °C^4,5^. From the result, the maximum carbon dioxide is absorbed through fermentation of 8.57 g by^6–8^. Recently, vegetable oils have been the potential source for highly replacing fossil fuel in the compression ignition engine. Preheated vegetable oil can be directly used in the engine than diesel fuel. Before using vegetable oil, the preheated process enhances the oil's performance and improves the engine’s performance^9,10^. Bioenergy is also termed zero waste. Generally, in zero waste, the organic wastes are converted into bioenergy. Most of the research is on utilising organic waste and producing effective biofuels.

Numerous organic wastes are available worldwide, and they can be converted into valuable fuel sources is one of the main tasks of scientists and researchers^11^. Many renewable sources are available, namely: biogas and coal. Bioethanol, biodiesel and biohydrogen. Organic wastes are dumped into the land; they can be needed for security, and maintaining their properties is a big challenge. The properties are changed due to climatic conditions^12^. Biofuel offers clean energy sources and better utilisation in all climatic conditions^13^. Due to the scarcity of current fuel, its high price, and its environmental impact, biofuel is one of the most promising alternative fuels around the globe. It will play a significant role in meeting the world’s energy demand.

Bioethanol production is attained through lignocellulosic biomass from vegetable species of the catatumbo area. Based on the study, the 2.301 × 10^7^m^3^ per year quantity of biogas is produced through the residue of onions and potatoes^14^. In bioenergy production the sorghum plays a significant role; different types of fuels are produced with the influence of sorghum. Bioenergy crops are vital in agriculture, human food, animal feed etc. Sorghum biomass such as stalks, leaves and grains are converted into liquid stages such as bioethanol, biodiesel, and bio-oil. It can be further turned into a gas such as biogas, biohydrogen etc. Different biomass pretreatment processes are available: biochemical, thermomechanical and biological^15,16^.

Bio-based catalysts are an excellent choice for biodiesel production and can also be replaced by a conventional chemical catalyst. Bio-based catalysts are comparatively low-cost and eco-friendly. Most biological, vegetable and restaurant wastes are converted into biocatalysts in biofuel production^17^. Using beef tallow with a co-solvent-based transesterification process offered radical biodiesel production. Biodiesel production is achieved using potassium hydroxide as a base catalyst, methanol as the primary solvent and ethanol as a co-solvent. Different process parameters involved in biodiesel production are oil/ alcohol ratio, methanol/ethanol ratio, catalyst concentration, reaction temperature and reaction time^18,19^.

The unanswered questions are: waste to fuel is excellent, but is there any potential to use biowaste as a catalyst to process and produce biodiesel from waste cooking oil? If so, what will be the yield of biodiesel? What are the influences of the molar ratio in such a process? This novel experimental approach uses waste vegetable oil from cooking and green plant waste to make biodiesel. To sustain diesel demand and environmental cleanup, biowaste catalysts were created from vegetable wastes, and biofuel was created from waste cooking oil. Various organic plant wastes, including bagasse, papaya stem, banana peduncle, and moringa oleifera, are heterogeneous catalysts for this research work. First, each plant waste is evaluated separately for its potential as a catalyst for biodiesel creation; next, all plant wastes are combined to create a single catalyst that is then used to produce biodiesel. The influences of the calcination temperature, reaction temperature, methanol/oil ratio, catalyst loading, and mixing speed were considered in the analysis of the maximum biodiesel yield.

The main objective of this work intense to maximise the biodiesel yield percentage with the assistance of organic plant wastes and used cooking oil. Other plant wastes are considered for this experimental work: bagasse, papaya stem, banana peduncle and moringa oleifera. Two stages of investigations are carried out. Initially, plant wastes are used individually to produce biodiesel further. All wastes are mixed and produce biodiesel. This team has expertise in fuel generation from various waste sources like waste cooking oil^20^, waste fish parts^21^ and waste transformer oil^22^. The novelty of this investigation is that marketplace waste like waste cooking oil and waste vegetable mixture is used for biodiesel production and thereby supports environmental degradation.

## Material and methods

Biofuel production is considered natural waste. This research takes various plant wastes and uses cooking oil to produce biodiesel. Plant wastes such as bagasse, papaya stem, banana peduncle and moringa oleifera are taken for this investigation and used cooking oil is considered for this investigation. In this investigation the plant's waste employed were, the waste which disposed in and around Koyambedu Wholesale Market Complex, koyambedu, Chennai, Tamil Nadu, India. It is declared that no samples collected from the wild or cultivation field by considering the national and international importance. All methods were performed in accordance with the relevant guidelines and regulations. The plant wastes are cleaned initially using running water further cleaning is conducted through distilled water^23^. During the completion of the cleaning process, the wastes are dried well with the assistance of sunlight for the one-week duration for extreme removal of water content present in the wastes. Initially, dried wastes were chopped into small pieces by cutting and ramming. Further, small pieces are independently converted into powder through a grinding process^24^. A sieving process separates the unwanted particles present in the powdered particles with ASTM 18 mesh size. The dispersion of liquid and gaseous products is significantly impacted by particle size, but the stability of the catalyst is also significantly impacted. While the gas selectivity decreased slightly for the 250–420 m catalyst, it reduced dramatically for the 40–60 and 60–100 m catalysts. This makes sense, given the significant butyraldehyde content of the big catalyst particles. Aldol-condensation processes facilitated by the zirconia support’s acid–base characteristics use the aldehyde as a precursor to deposits, which deactivate the catalyst^25,26^. Hence unwanted materials were visibly not separated, or with the help of hand, the mesh 18 was selected to limit the size to 1000 µm.

Usually, agro -Waste based catalyst demands high calcination temperature for higher yield^27^, prepared biodiesel with 100% yield from Tectona grandis Leaves at 700 °C calcination temperature, 4 h at Methanol/oil ratio 6:1 with catalyst loading was 2.5%. Similarly, many researchers achieved higher yields at the same calcination temperature of 700 °C for 4 h^28^ used Methanol/oil Ratio 7.6:1 wt% catalyst loading to extract biodiesel from Banana peels and achieved 98.5% yield^29^. Extracted biodiesel from Ripe Plantain peels with a Methanol/oil Ratio of 0.73 v/v catalyst loading and produced a yield of 99.2%^30^. Produced biodiesel from Red banana Peduncle with a yield of 98.69% using a Methanol/oil Ratio of 9.20 wt% catalyst loading. Cocoa pod husk was employed by^31^ to extract biodiesel with a yield of 99.3% using a Methanol/oil Ratio of 7.6:1 and 0.73 v/v catalyst loading^32^. Extracted biodiesel from Carica papaya Peels with a Methanol/oil Ratio of 12:1 at the catalyst loading of 3.5 wt% and produced a yield of 97.5%.

**Figure 1:**
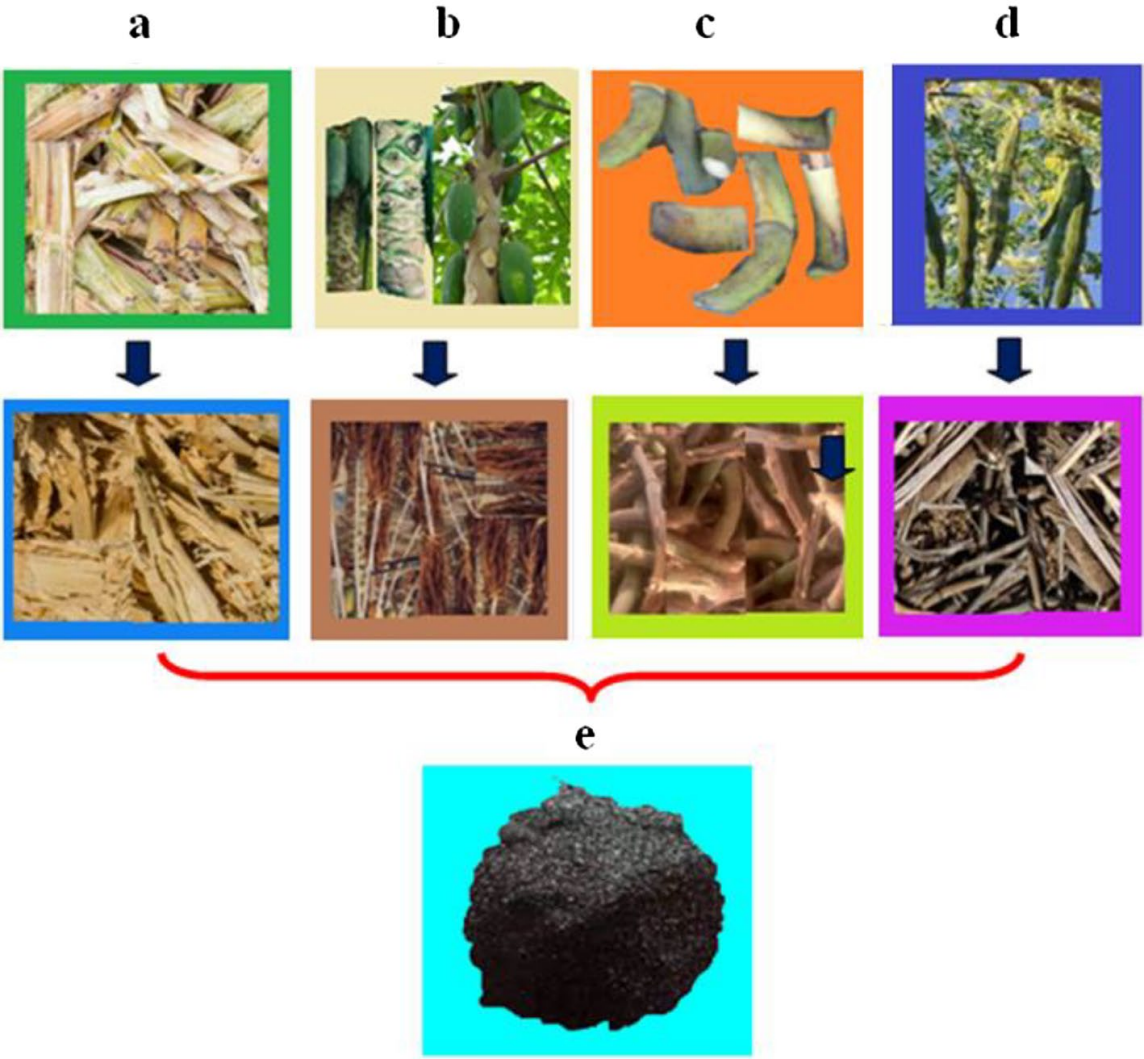
Multi-plant wastes before and after drying: (**a**) Bagasse (**b**) Papaya stem (**c**) Banana peduncle (**d**) Moringa oleifera and (**e**) Calcined plant waste ash.

Calcinated plant wastes are stored separately for further use in biodiesel preparation^33,34^. Thus All plant waste sieved powders are recommended to calcinate through a muffle furnace and maintain at 700 °C for 4 h. The standard transesterification time upto120 minutes is followed for all kinds of biodiesel production. Figure 1 illustrates the plant waste before and after dried conditions and calcinated plant waste ash.

This investigation identified that the vegetable waste found nearby the marketplaces is a promising source of catalysts for biodiesel production from various natural resources. Based on their experimental findings^35^, suggested that spent sugarcane bagasse be made into a solid catalyst that could be reused, inexpensive, and utilised to make biodiesel. In addition to developing alternative materials for sustainable applications, they emphasised that waste sugarcane bagasse can be used as a low-cost base catalyst for biodiesel production on a wide scale. Waste Carica Papaya stem was suggested by^36^ to make biodiesel from used cooking oil and Scenedesmus obliquus lipid using a hydrophobic solid base catalyst. They said, “This process is an energy-efficient alternative to the published methods due to the reusable green heterogeneous catalyst utilised, the substrate tolerance, the speed of the reaction, and the high yield^37^ recommended a red banana peduncle-based catalyst at high temperatures and its use in the transesterification of Ceiba pentandra oil for biodiesel production to create a unique heterogeneous base catalyst. To construct a particular heterogeneous base catalyst, they suggested using a red banana peduncle-based catalyst at high temperatures and using it to transesterify Ceiba pentandra oil to generate biodiesel. A heterogeneous catalyst made from moringa leaves was the subject of experiments by^38^. All the above investigators were proved through their detailed characterisation of the developed catalyst by employing EDX, X-EDS, SEM and XRD. Hence this is not focused on characterisation. In such a waste mixture, separating each kind of waste for catalyst preparation is very tedious. The mixture quantity may not be equal. In practical cases, the mixture of vegetable waste may not have an equal share, so characterisation may not require as we cannot assure reliable values. But all the promising sources give good outcomes. According to the aim of sustainable development of a catalyst for biodiesel production (SDG 9) and environmental remediation (SDG 3). In this investigation, are there any potential possibilities to improve the yield or obtain an equivalent yield by developing a catalyst from mixed vegetable waste? But for experimentation purposes, an equal quantity of waste was considered.

## Experimental procedure

An experimental investigation is conducted through three-necked glass flask arrangements; a flask having a round shape in the bottom area accumulates up to 1000 ml of testing liquid medium^34^. An inbuilt condenser arrangement is provided in the vertical direction. The magnetic stirrer with inbuilt hot plate apparatus is used for effective biodiesel production. The model of the apparatus is Model: Q-19A, Length: 9 × 35 mm, Magnetic Stirrer, 150 × 220 × 150 mm Aluminium Section 2 ltr capacity ^39^.

Waste vegetable oil must undergo pre-treatment to be used in the transesterification process because its free fatty acids can cause soap to form when they react with the alkaline catalyst. Initially, the used cooking oil is preheated at 40 °C and 50 min homogeneously, excluding methanol and plant waste ash catalyst. A sample of 100 ml of used cooking oil is poured into the reactor unit for the transesterification process with the preheating process^40^. After preheating, adding of alcohol and plant waste catalyst into the flask. Maintain and ensure the proper mixing of MeOH and oil. The mixing speed is maintained for the chosen levels. After the reaction, the mixture cools and transforms into a separating funnel^41^. Three different layers are attained: the upper layer composed of Fatty Acid Methyl Ester, the middle layer glycerine and the bottom layer occupied by the catalyst. Hence the liquid components were placed directly into glass separation funnels and the solid particles were meticulously separated by centrifugation. Further, the mixture is washed and dried to obtain biodiesel. Different process parameters are considered for the conduction of the transesterification process are calcination temperature (350 °C, 400 °C, 450 °C, 500 °C, 550 °C and 600 °C), reaction temperature (40 °C, 50 °C, 60 °C, 70 °C, 80 °C and 90 °C), methanol/oil ratio (6:1, 9:1, 12:1, 15:1, 18:1 and 21:1), catalyst loading (2 wt%, 2.5 wt%, 3.0 wt%, 3.5 wt%, 4.0 wt%, 4.5 wt% and 5.0 wt%) and mixing speed (350 rpm, 400 rpm, 450 rpm, 500 rpm, 550 rpm, 600 rpm and 650 rpm). The atmospheric pressure level is maintained for conducting all experiments^42,43^. Simply the biodiesel yield is examined by the following formula in Eq. (1)

1 $${\text{Biodiesel yield }} = \, \left( {{\text{Quantity of biodiesel produced}}/{\text{quantity of oil used}}} \right) \, \times { 1}00.$$

## Results and discussion

Typically biofuel, such as biodiesel production, was concentrated on different parameters and the parameters highly influenced biofuel production^44^. This experimental investigation considered various parameters: calcination temperature °C, the reaction temperature in degree centigrade, methanol/oil ratio, catalyst loading (wt%) and mixing speed (rpm). These parameters were selected with different levels for attained a higher biodiesel yield percentage.

### Effects of calcination temperature

A novel study of various plant wastes was utilised to maximise biodiesel production. This analysis involved various plant wastes to maximise the biodiesel yield percentage. Compared to different plant wastes such as bagasse, papaya stem, banana peduncle and moringa oleifera, the mixed plant waste offered a higher biodiesel yield percentage, such as 89%, with 550 °C of calcination temperature. Conversely, the lower calcination temperature (350 °C) with banana peduncle waste recorded a lower biodiesel yield % (59%). Other plant wastes and calcination temperature effects are presented in Table 1.

**Table 1:** Calcination temperature effects in biodiesel yield.

Biodiesel yield in %					
Calcination temperature ^o^C	Bagasse	Papaya stem	Banana peduncle	Moringa oleifera	Mixed waste
350	60	63	59	68	78
400	67	70	64	73	81
450	71	74	74	81	84
500	75	78	82	85	86
550	77	84	83	87	89

**Figure 2:**
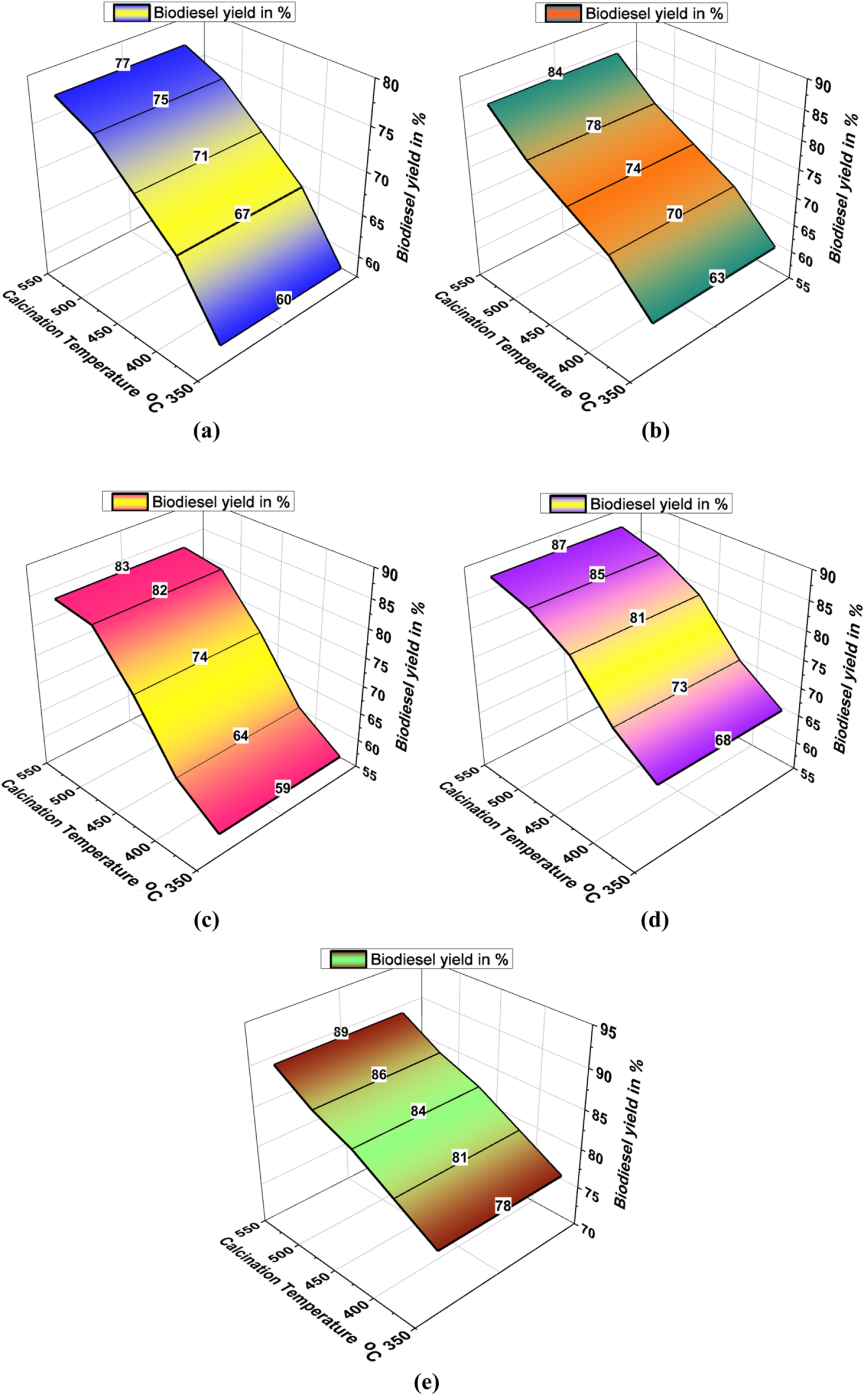
Calcination temperature vs. biodiesel yield in % using of plant waste: (**a**) Bagasse (**b**) Papaya stem (**c**) Banana peduncle (**d**) Moringa oleifera (**e**) Mixed waste.

Figure 2a illustrates the influence of calcination temperature on the biodiesel yield percentage using Calcinated bagasse waste. A higher biodiesel yield % was obtained at 77% at maximum calcination temperature (550 °C); contrary, the minimum biodiesel yield was achieved at 60% by using 350 °C of calcination temperature. Figure 2b shows the minimum and maximum biodiesel yield percentage using papaya stem waste Calcinated ash. This analysis recorded maximum and minimum biodiesel yield percentages as 84% and 63%, respectively. Higher calcination temperature offered maximum biodiesel yield percentage. Figure 2c presents the Banana peduncle calcinated ash influences in biodiesel yield percentage, for increased calcination temperature increases the biodiesel yield %^45^. In this analysis, the maximum biodiesel yield was achieved as 83%. Similarly, increasing calcination temperature increases the biodiesel yield % as shown in Fig. 2d.

Higher biodiesel yield was attained at 87% using Moringa oleifera waste calcinated ash. In all plant waste calcinated ash, the mixed waste calcinated ash offered a higher biodiesel yield percentage, such as 89%, as shown in Fig. 2e. From all plant waste utilisation, 550 °C of calcination temperature produced a maximum level of biodiesel yield %.

**Figure 3:**
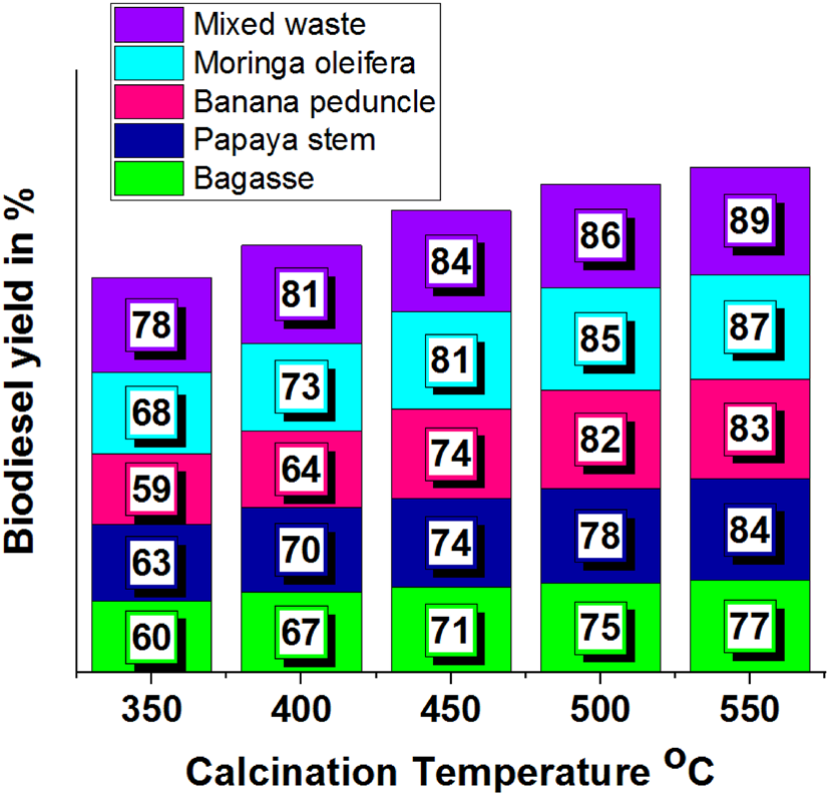
Comparison of biodiesel yield with different plant waste.

Figure 3 demonstrates the biodiesel yield percentage with the influence of different calcinated plant waste ash at different calcination temperatures. For this comparison analysis, mixed waste ash produced a maximum biodiesel yield percentage (89%) at maximum calcination temperature^36,41^.

### Effects of reaction temperature

Influencing reaction temperature, the minimum and maximum biodiesel yield % were recorded as 60% and 88% (Table 2). Maximum biodiesel yield was offered by mixed waste, and minimum biodiesel yield was attained by banana peduncle calcinated ash.

**Table 2:** Reaction temperature effects in biodiesel yield.

Biodiesel yield in %					
Reaction temperature °C	Bagasse	Papaya stem	Banana peduncle	Moringa oleifera	Mixed waste
40	61	63	60	65	78
50	66	69	71	70	81
60	72	75	77	79	85
70	76	79	83	84	86
80	77	85	82	87	88
90	74	77	75	83	85

**Figure 4:**
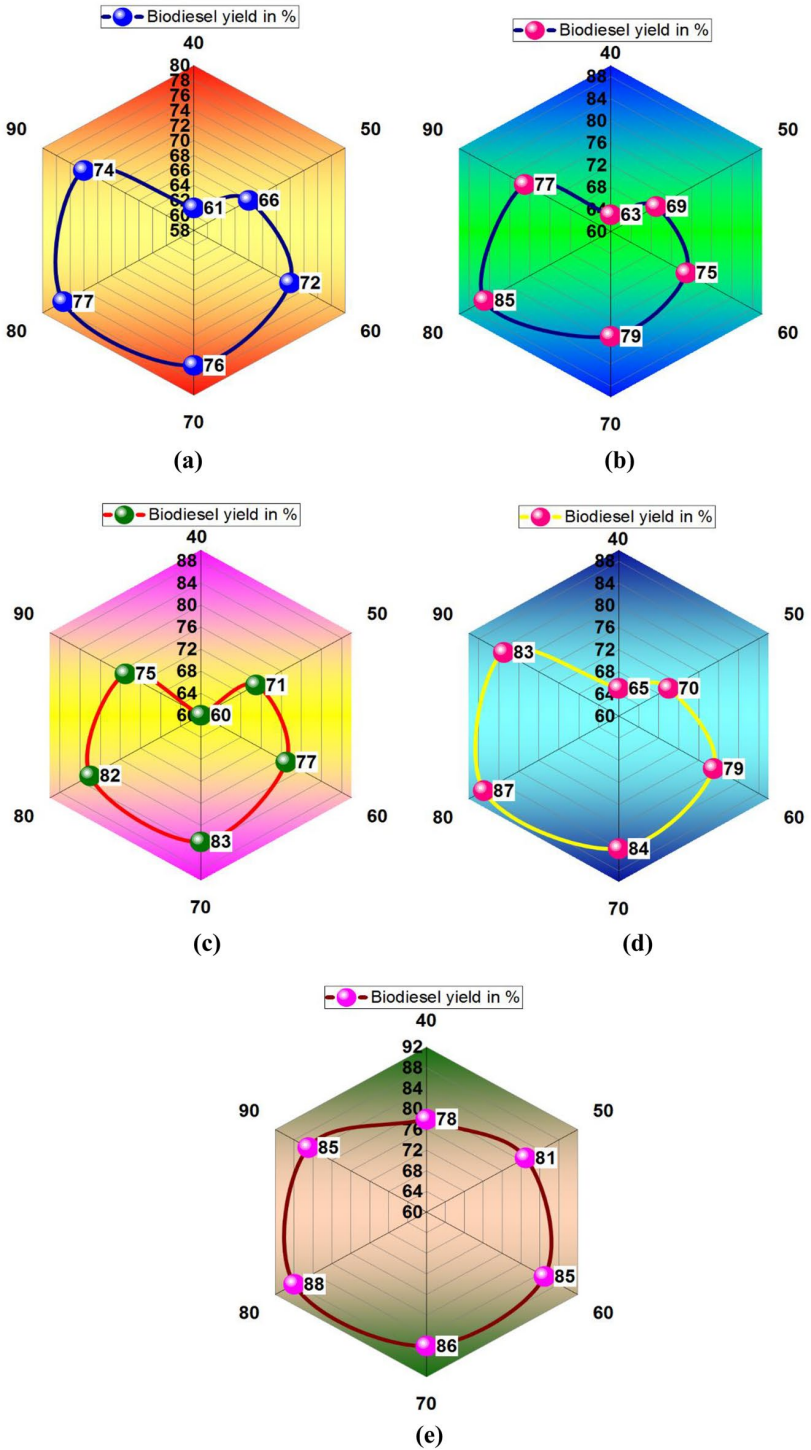
Reaction temperature vs. biodiesel yield in % using of plant waste: (**a**) Bagasse (**b**) Papaya stem (**c**) Banana peduncle (**d**) Moringa oleifera (**e**) Mixed waste.

Figure 4 shows that another plant waste increases biodiesel yield percentage. Figure 4a exemplified that a higher biodiesel yield (77%) was attained through bagasse waste ash with 80 °C of reaction temperature^46,47^. Using of papaya stem ash, the maximum biodiesel yield was received as 85% by 80 °C of reaction temperature, as shown in Fig. 4b.

A reaction temperature of 70 °C with banana peduncle waste ash offered 83% of the biodiesel yield percentage, as shown in Fig. 4c. Moringa oleifera plant waste ash offered 87% biodiesel yield with 80 °C of reaction temperature, illustrated in Fig. 4d. In all plant waste ash involvement, the mixed plant waste provided a higher yield of 88% at 80 °C of reaction temperature, as shown in Fig. 4e.

**Figure 5:**
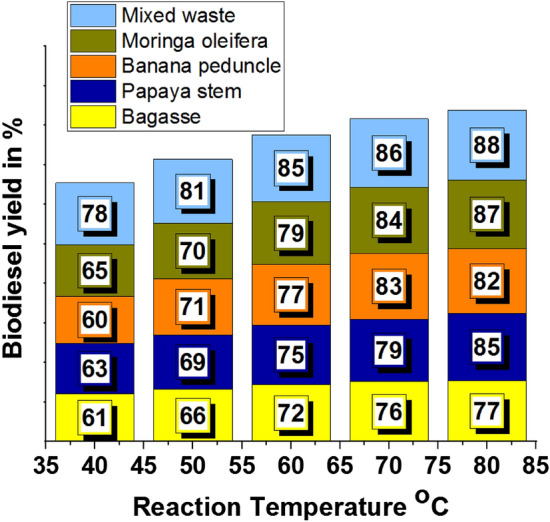
Comparison of biodiesel yield with different plant waste (reaction temperature).

Figure 5 shows the comparative analysis of biodiesel yield percentage by different plant waste ash concentrations. In contrast to all waste ash, the mixed waste ash recorded a maximum yield such as 88%, which proves the mixed waste ash has high calcination properties. Keeping the methanol to oil ratio at 18:1^48^, studied the impact of reaction temperature (40 to 80 °C) on biodiesel yield. 2 wt% of Nanocatalyst was used, and the reaction took place for 2 h. For biodiesel production, they utilised MgO-SnO_2_ Nanocatalysts with increased surface area^49^. A maximum of 81.1% biodiesel yield was reported at 110 °C with catalyst loading of 7 wt%, agitation speed of 600 rpm, methanol to oil ratio of 30:1, and reaction period of 270 min when the process parameters for the production of biodiesel from used cooking oil in the presence of this catalyst were optimised^50^.

### Effects of methanol/oil ratio

The methanol/oil ratio influencing minimum and maximum biodiesel yield is presented in Table 3. From that analysis, a methanol/oil ratio of 18 offered a higher biodiesel yield, such as 92%, contrary minimum yield of 75%, by a methanol/oil ratio of 6.

**Table 3:** Methanol/oil ratio effects in biodiesel yield.

Biodiesel yield in %					
Methanol/oil ratio	Bagasse	Papaya stem	Banana peduncle	Moringa oleifera	Mixed waste
6	75	78	75	77	82
9	79	80	79	80	83
12	82	84	83	84	88
15	86	88	87	86	90
18	87	89	89	90	92
21	83	85	86	83	88

**Figure 6:**
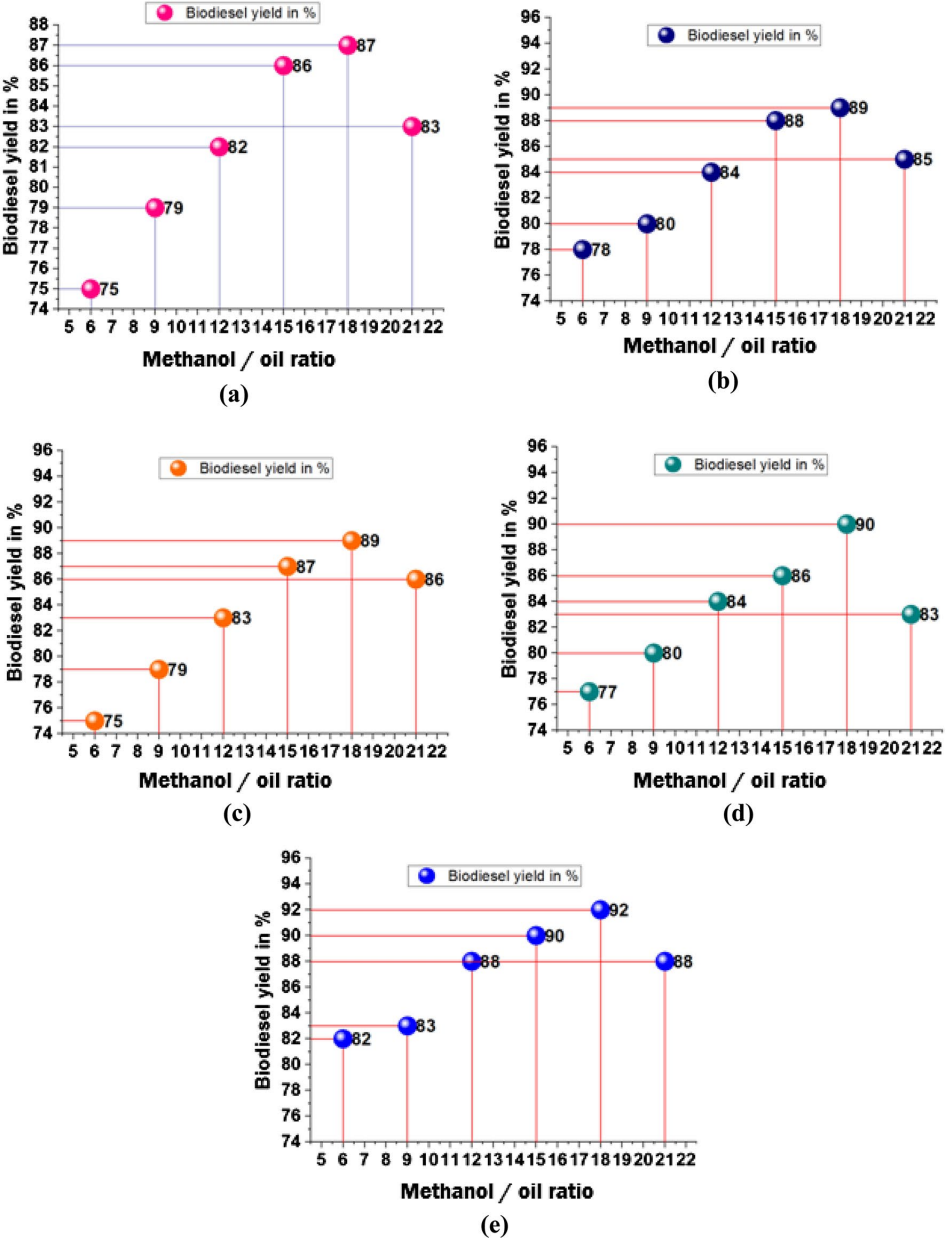
Methanol /oil ratio vs. biodiesel yield in % using of plant waste: (**a**) Bagasse (**b**) Papaya stem (**c**) Banana peduncle (**d**) Moringa oleifera (**e**) Mixed waste.

Increasing of methanol/oil ratio increases the biodiesel yield, further increasing the yield was slowly reduced, as shown in Fig. 6. The higher yield was registered as 87% by influencing the methanol/oil ratio of 18 with bagasse waste ash illustrated in Fig. 6a. Employing papaya stem ash, with a methanol/oil ratio of 18, was recorded as 89% of biodiesel yield, as shown in Fig. 6b. Similarly, banana peduncle waste ash offered 89% of the biodiesel yield percentage, as shown in Fig. 6c. Moringa oleifera plant waste ash increases the biodiesel yield percentage, such as 90%, with an 18-methanol/oil ratio, illustrated in Fig. 6d. Mixed plant waste ash offered a maximum yield of 92%, as shown in Fig. 6e.

**Figure 7:**
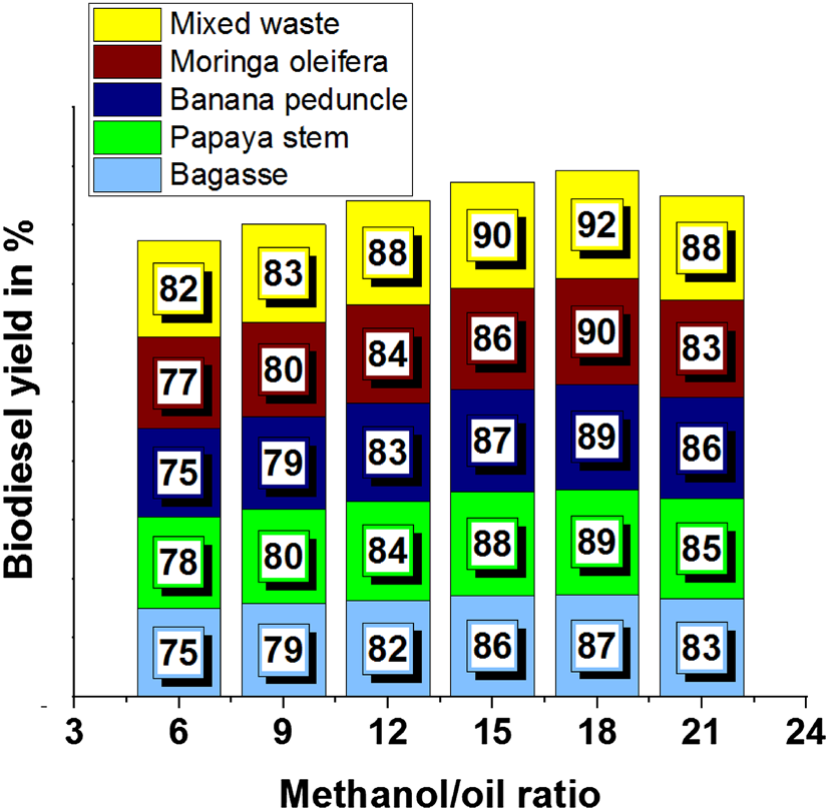
Comparison of biodiesel yield with different plant waste (methanol/oil ratio).

The minimum biodiesel yield was observed as 75% and the maximum as 92%, as shown in Fig. 7. Increasing the methanol/oil ratio concentration increased the yield constantly; increasing the methanol/oil ratio from 18 to 21 decreased slightly. Compared to all plant waste, bagasse waste produced a minimum yield percentage^51^ reported that a lower yield of 66.2% biodiesel was observed. This is the case due to the difficulty of glycerol separation at more excellent molar ratios than 12:1. A fraction of the glycerol remained in the alkyl esters phase, which also decreased the yield of alkyl esters.

Ref.^52^The mole ratio of methanol to oil was increased 7 times, which promoted the forward reaction for biodiesel production and resulted in the highest biodiesel yield (88.1 1.5%). However, as the mole ratio of methanol was increased further, the output of biodiesel declined from 88.1 1.5% of 1:7 (oil/methanol) to 49.0 0.8% of 1:12; this was due to the presence of too much methanol in the reaction system, which can reduce enzyme activity. In earlier publications^34^, methanol was shown to have a comparable inhibitory impact on lipase activity.

### Effects of catalyst loading (wt%)

Increasing catalyst loading increased the biodiesel yield percentage, as presented in Table 4. Minimum and maximum levels of biodiesel yield were recorded as 78% and 95%, respectively. Using 4.5 wt% of catalyst loading offered maximum biodiesel yield even in all plant waste usage.

**Table 4:** Catalyst loading (wt %) effects on biodiesel yield.

Biodiesel yield in %					
Catalyst loading (wt %)	Bagasse	Papaya stem	Banana peduncle	Moringa oleifera	Mixed waste
2	78	80	79	82	84
2.5	84	85	85	86	88
3	85	86	87	88	90
3.5	87	88	88	89	91
4	88	90	89	93	94
4.5	89	90	90	92	95
5	87	86	88	89	92

**Figure 8:**
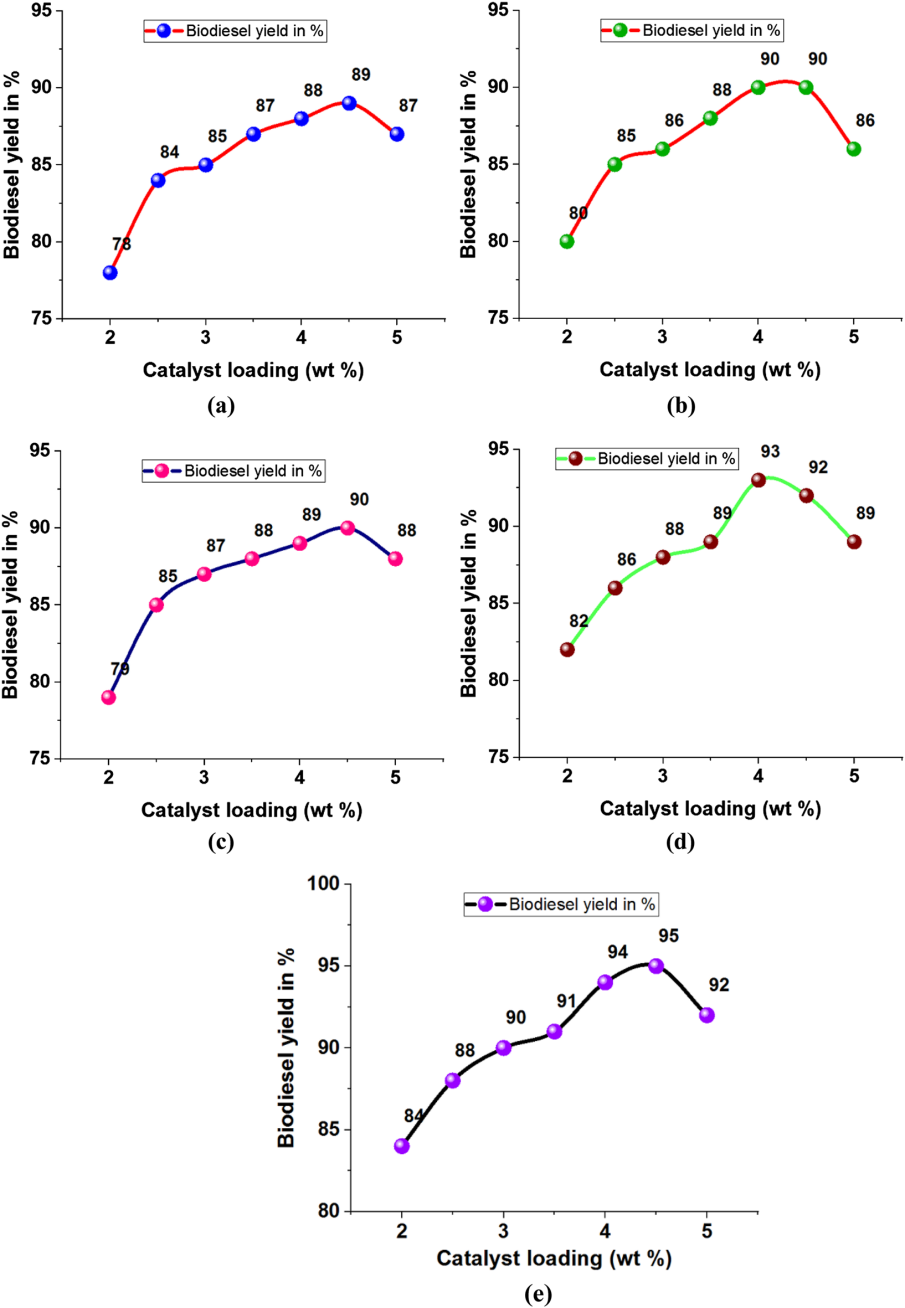
Catalyst loading (wt%) vs. biodiesel yield in % using plant waste: (**a**) Bagasse (**b**) Papaya stem (**c**) Banana peduncle (**d**) Moringa oleifera (**e**) Mixed waste.

A maximum biodiesel yield of 89% was registered using bagasse waste ash with 4.5 wt% of catalyst loading, as shown in Fig. 8a. Similarly, using papaya stem and banana peduncle waste ash with 4.5 wt % of catalyst loading produced 90% of biodiesel yield, as shown in Fig. 8b and Fig. 8c. Using moringa oleifera waste ash with 4 wt % of catalyst loading recorded 93% of biodiesel yield as shown in Fig. 8d. Other mixed waste ash with 4.5 wt % of catalyst loading provided a maximum yield percentage of 95%, as shown in Fig. 8e.

**Figure 9:**
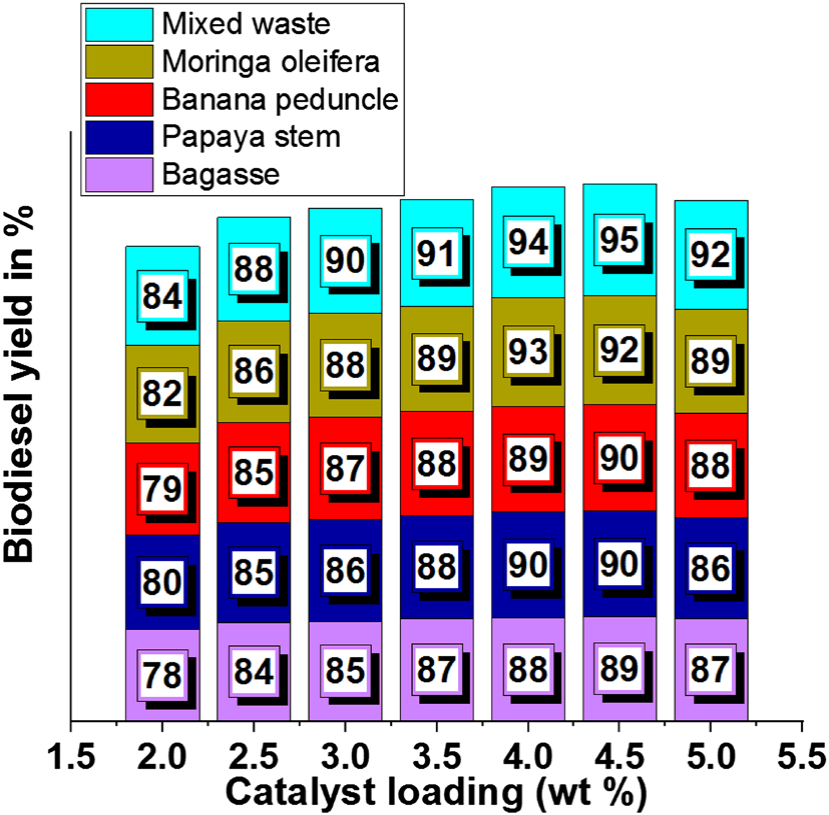
Comparison of biodiesel yield with different plant waste (Catalyst loading).

The influence of catalyst loading 5 wt% was changed in the biodiesel yield percentage shown in Fig. 9. Initially, the catalyst loading of 2 wt% offered a minimum level of biodiesel yield. Further increasing catalyst loading to 4.5 wt% provided the maximum yield percentage. Mixed waste ash provided a higher yield percentage.

### Effects of mixing speed

Mixing speed is one of the influencing parameters in the biodiesel yield percentage; Table 5 presents the different mixing speeds and yield percentages. Mixed waste ash with 550 rpm mixing speed offered an excellent yield percentage, such as 99%, compared to other waste ash contributions.

**Table 5:** Mixing speed (rpm) effects in biodiesel yield.

Biodiesel yield in %					
Mixing speed (rpm)	Bagasse	Papaya stem	Banana peduncle	Moringa oleifera	Mixed waste
350	90	92	91	93	94
400	92	93	92	94	96
450	93	94	95	96	97
500	94	95	95	97	98
550	95	96	96	97	99
600	96	96	97	98	99
650	93	95	94	95	97

**Figure 10:**
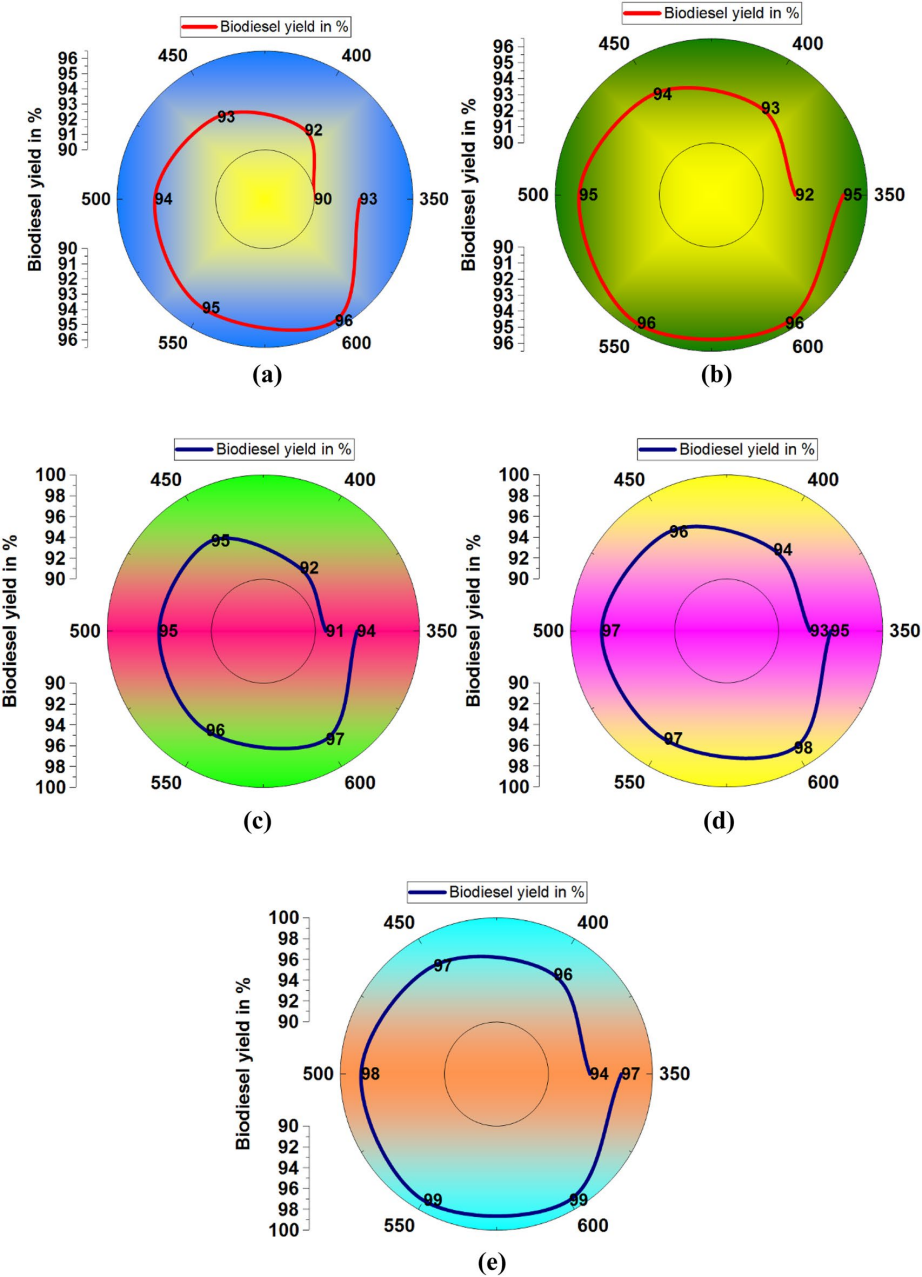
Mixing speed (rpm) vs. biodiesel yield in % using of plant waste: (**a**) Bagasse (**b**) Papaya stem (**c**) Banana peduncle (**d**) Moringa oleifera (**e**) Mixed waste.

Figure 10 illustrates the mixing speed influence with various plant waste ashes for increasing of biodiesel yield. Figure 10a shows that involving bagasse waste in the biodiesel yield, 600 rpm of mixing speed offered 96% of biodiesel yield. Similarly, papaya stem waste produced 96% of biodiesel yield at 600 rpm mixing speed, as shown in Fig. 10b. Banana peduncle waste recorded 97% of the yield, and moringa oleifera waste registered 98% yield, shown in Fig. 10c and d, respectively. A higher yield of 99% occurred at 550 rpm and 600 rpm of mixing speed, respectively. Mixed waste produced a maximum yield (99%) with an influence of 600 rpm mixing speed.

**Figure 11:**
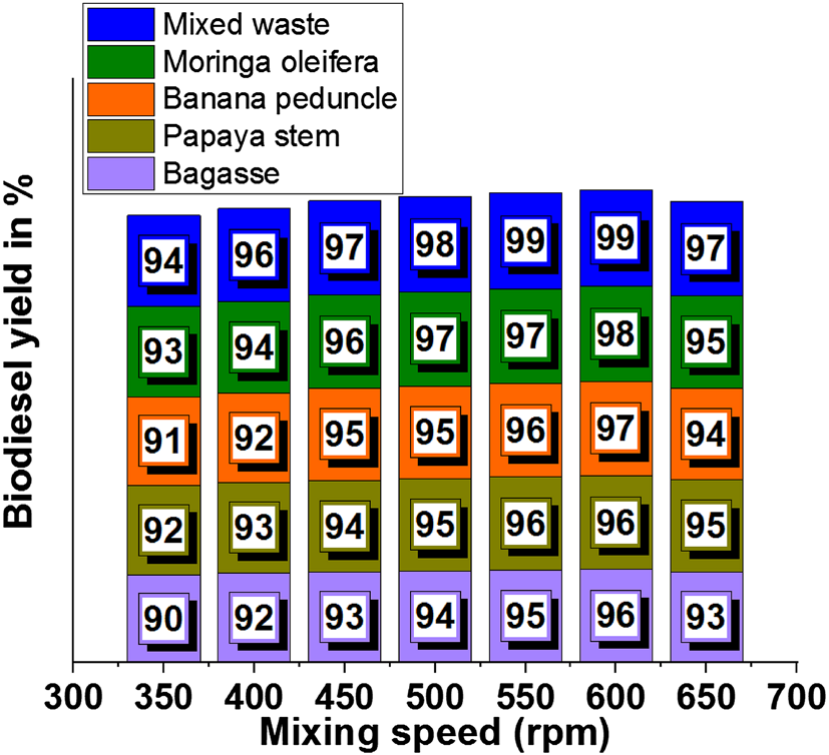
Comparison of biodiesel yield with different plant waste (mixing speed).

Figure 11 presents the comparative analysis of various plant wastes influenced by the biodiesel yield with mixing speed. All the plant wastes produced more than 90% of biodiesel yield with the effects of different mixing speeds. Above 500 rpm of mixing speed influenced to production of more than 95% of biodiesel yield.

Waste Carica Papaya stem created a hydrophobic solid base catalyst that produced biodiesel from used cooking oil and Scenedesmus obliquus lipid. The catalyst demonstrated the highest efficiency with a maximum fatty acid methyl ester conversion of 95.23% for used cooking oil and 93.33% for Scenedesmus obliquus lipid within 3 h. Furthermore, the disclosed method using CCPS tolerates various aryl aldehydes and produces the appropriate BMN derivatives in significant yields. The reusability analysis revealed that even after five repeated cycles, the catalyst maintained good catalytic activity for the generation of biodiesel (i.e. conversion > 85%) and Knoevenagel condensation. Because of the reusable green heterogeneous catalyst used, the substrate's tolerance, the reaction’s speed, and the high yield, this procedure is more energy-efficient than the reported methods^42^. Reported about his experimental results that Waste sugarcane bagasse was converted into a reusable, affordable solid catalyst used in biodiesel production. According to the analysis, the catalyst comprises several metal oxides and carbonates. This catalyst, which was polycrystalline and micro-mesoporous, had a low surface area of 7.66 m2 g1. In 285 min, the catalyst produced 92.84 weight percent of biodiesel at 9:1 MTOR, 10 weight percent catalyst, and 65 °C. The catalyst has good efficacy for the reaction with TOF of 6.59 h1 and Ea value of 22.12 kJ mol1, and it is essential with fundamental strength of 10.1 H_ 18.4. The reaction was shown to be endothermic and non-spontaneous by the thermodynamic analysis. Waste sugarcane bagasse has the potential to be developed as a low-cost base catalyst for biodiesel production on a large scale, along with the development of other materials for sustainable applications, due to its abundant availability, renewability, and high economic feasibility.

Waste Carica Papaya stem was used by^32^ to create a hydrophobic solid base catalyst that produced biodiesel from used cooking oil and Scenedesmus obliquus lipid. The catalyst demonstrated the highest efficiency with a maximum fatty acid methyl ester conversion of 95.23% for used cooking oil and 93.33% for Scenedesmus obliquus lipid within 3 h. Furthermore, the disclosed method using CCPS tolerates various aryl aldehydes and produces the appropriate BMN derivatives in significant yields. The reusability analysis revealed that even after five repeated cycles, the catalyst maintained good catalytic activity for the generation of biodiesel (i.e. conversion > 85%) and Knoevenagel condensation. Because of the reusable green heterogeneous catalyst used, the substrate's tolerance, the reaction's speed, and the high yield, this procedure is more energy-efficient than the reported methods^37^. Developed red banana peduncle-based catalyst at high temperatures and used in the transesterification of Ceiba pentandra oil for biodiesel production to create a unique heterogeneous base catalyst. According to the characterisation results, the minerals are dramatically extracted from the peduncle by the chosen calcination temperature (700 °C for 4 h), degrading the carbohydrate-lignin matrix. Due to its larger surface area and mixed mineral oxides, the synthesised calcined red banana peduncle catalyst demonstrated a high catalytic activity level. The transesterification parametric effect on C. pentandra methyl esters conversion was investigated using central composite design-based response surface methodology. It was discovered that the chosen quadratic model was significant. Under the ideal conditions of 2.68 weight percent calcined red banana peduncle concentration, 11.46:1 methanol to esterified Ceiba pentandra oil molar ratio, and 106 min of reaction time, the model predicted a maximum C. pentandra methyl esters conversion of 99.23%. To produce a sustainable preparation for biodiesel^38^, concentrated on creating a heterogeneous catalyst from moringa leaves. The catalyst was made by simple calcination at 500 °C for two hours, and it was then used immediately in a transesterification reaction using oil and methanol to make biodiesel. Following calcination, dolomite, calcite, and (K_2_Ca(CO_3_)_2_) inorganic carbonate minerals were produced, and these minerals aid in the transesterification reaction, according to the results of characterisation. As a result, an 86.7% FAME yield was seen under the following reaction conditions: 120 min, 65 °C, 6 wt% catalyst dosage, and a 6:1 methanol/oil molar ratio. Hence the catalyst was recommended to prepare from mixed vegetable waste at 700 °C and verified the biodiesel yield.

## Conclusions

This experimental work was chosen plant waste ash and cooking oil for biodiesel production and successfully increased the biodiesel yield percentage. Initially, plant wastes were independently used further. All wastes were mixed well and used to increase biodiesel yield percentage effectively. The novelty of this work produced better results as follows:

- From the calcination temperature effects, the bagasse ash produced a minimum of 60% and a maximum of 77% biodiesel yield at 350 °C and 550 °C, respectively. In similar calcination temperature levels, the papaya stem waste ash produced 63% and 84%, respectively, followed by banana peduncle 59% and 83%, respectively. Moringa oleifera ash offered 68% and 87%, respectively, and mixed waste ash produced higher yields of 78% and 89%, respectively.
- Influencing of reaction temperature, the higher yield was recorded as using of bagasse ash (77%), papaya stem (85%), banana peduncle (82%), moringa oleifera (87%) and mixed waste (88%) with 80 °C of reaction temperature. Involving of Methanol/Oil ratio extreme yield was achieved as 87% produced by bagasse ash, 89% of papaya stem, 89% of the banana peduncle, 90% of moringa oleifera and 92% of mixed waste.
- More than 90% of biodiesel yield was achieved by involving all plant wastes independently and mixed with the support of 4.5 wt% of catalyst loading. Mixing speed is highly involved in producing more than 95% biodiesel yield while utilising all plant wastes. Among all plant wastes, mixed waste produced a high yield percentage even in all parameters influenced by biodiesel production.
- The novelty of this experimental work was the hope for the production of biodiesel massively with the assistance of organic plant wastes.

## Data Availability

The datasets generated during and/or analysed during the current study are available from the corresponding author and can be shared on reasonable request.
